# Lipid management in high cardiovascular risk patients in France: a comparison with rest of Europe patients from 1-year follow-up of SANTORINI

**DOI:** 10.3389/fcvm.2026.1748457

**Published:** 2026-03-12

**Authors:** Jean Ferrières, Kausik K. Ray, Alberico L. Catapano, Radhouan Allouche, Christian Becker, Aurélie Bardet, Michel Farnier

**Affiliations:** 1 Department of Cardiology and INSERM UMR 1295, Toulouse Rangueil University Hospital, Toulouse University School of Medicine, Toulouse, France; 2 Imperial Centre for Cardiovascular Disease Prevention, ICTU-Global, Imperial College London, London, United Kingdom; 3 Department of Pharmacological and Biomolecular Sciences, University of Milan, Milan, Italy; 4 Department of Pharmacological and Biomedical Sciences, University of Milan Multimedica IRCCS, Milan, Italy; 5 Daiichi Sankyo Europe GmbH, Rueil-Malmaison, France; 6 Daiichi Sankyo Europe GmbH, Munich, Germany; 7 PEC2 team, UR7460, University of Burgundy Europe, Dijon, France

**Keywords:** cardiovascular risk, cohort study, combination therapy, lipid-lowering therapy, low-density lipoprotein cholesterol, observational

## Abstract

**Background:**

The SANTORINI study (NCT04271280) was designed to assess lipid management in different European countries and care settings over 1 year of follow-up. Here, we report prospective findings of patient characteristics, low-density lipoprotein cholesterol (LDL-C) goal attainment, and treatment patterns of lipid-lowering therapies (LLTs) in France and the rest of Europe (RoE; without France) at 1-year follow-up.

**Materials and methods:**

Patients at high or very high cardiovascular (CV) risk were recruited across 14 European countries from 17 March 2020 to 11 February 2021 and followed for 1 year of prospective follow-up.

**Results:**

Among 9,559 patients enrolled in the study, 8,802 with available risk classification data and 7,210 with available LDL-C data at baseline and 1-year follow-up were included. Of patients with available LDL-C data, 621 were included in the France cohort and 6,589 were included in the RoE cohort. In the France cohort (full analysis set for risk), investigators classified 20.1% and 79.9% of patients as high and very high CV risk, respectively. When CV risk was recalculated centrally as per the 2019 European Society of Cardiology/European Atherosclerosis Society guidelines, 7.1% and 92.9% of patients in the France cohort were considered high and very high risk, respectively. Total LLT monotherapy and combination therapy use over 1 year increased from 51.2% to 58.5% and 25.3% to 38.2%, respectively. Overall, mean (standard deviation) LDL-C level decreased from 2.6 (1.3) mmol/L to 2.2 (1.2) mmol/L, and LDL-C goal attainment increased from 14.8% to 23.4%. Results in the France cohort were consistent with those observed in the RoE cohort.

**Conclusions:**

While use of combination LLT and LDL-C goal attainment increased in the France cohort over 1 year of follow-up, the majority of patients remained at an increased risk of CV events.

## Introduction

Cardiovascular (CV) disease is the second leading cause of death in France (1). It is well established that elevated low-density lipoprotein cholesterol (LDL-C) is associated with an increased risk for atherosclerotic cardiovascular disease (ASCVD), and a 1 mmol/L reduction in LDL-C level leads to a 22% reduction in the risk for major CV events (2). As such, LDL-C is a central target for the prevention of ASCVD.

In 2019, the European Society of Cardiology (ESC) and the European Atherosclerosis Society (EAS) updated their joint guidelines to recommend more stringent LDL-C goals, particularly for patients at high (<1.8 mmol/L) and very high (<1.4 mmol/L) CV risk (3). Despite the availability of several lipid-lowering therapies (LLTs) and evidence-based treatment guidelines, LLT usage and LDL-C goal achievement are suboptimal across Europe, including France (4–6).

Data from the Esteban study, a cross-sectional, publicly funded survey conducted in 2015, showed that hypercholesterolemia affected approximately 23% of the French population (4). The proportion of patients in secondary care receiving LLT was 29.7% (4). Similarly, the DAUSSET study, a national, multicenter, non-interventional study, reported that only 41.7% of patients achieved an LDL-C goal of <70 mg/dL (1.8 mmol/L), underscoring the persistent challenge of lipid control in very high-risk populations, despite the availability of LLTs and guideline recommendations (7).

The SANTORINI (Treatment of High and Very High ri**S**k Dyslipidemic p**A**tients for the Preve**NT**ion of Cardi**O**vascula**R** Events in Europe—a Mult**I**natio**N**al Observat**I**onal Study) study (NCT04271280) was a European observational study that recruited patients at high and very high CV risk, and was designed to assess whether lipid management changed in European clinics following publication of the 2019 ESC/EAS dyslipidemia guidelines (6). Over 1 year of follow-up, an increase in the intensity of LLT regimens was observed across Europe, which resulted in an improvement in the proportion of patients achieving their risk-based LDL-C goals. Nevertheless, two-thirds of patients did not achieve their risk-based LDL-C goals and CV events remained high (6).

Here, we aim to report patient characteristics, treatment patterns of LLTs, LDL-C levels, and goal attainment in the SANTORINI France cohort at 1-year follow-up to assess whether the implementation of lipid management strategies had improved in the year following the publication of the 2019 ESC/EAS dyslipidemia guidelines. We also provide a descriptive comparison with the rest of Europe (RoE; without France) cohort observed in SANTORINI.

## Materials and methods

### Study design and population

SANTORINI was a prospective, observational study that enrolled patients aged 18 years or older who were at high or very high CV risk across 14 European countries. The rationale and methods used in SANTORINI have been described previously (8).

Patients requiring LLTs were recruited from 17 March 2020 to 11 February 2021 and followed for 1 year of prospective follow-up. The final database lock date was 31 May 2022. CV risk was assigned by the investigators at enrollment, and the basis for risk classification was documented. At baseline, patients' characteristics and medical history were documented, as well as LLTs and other co-medications. Risk was assigned by the investigators, but risk was also calculated during the analysis of the registry data based on the documented patient information (SMART, Framingham, and SCORE risk score systems—including the SCORE2 system analyzed similarly to SCORE).

At the 1-year follow-up visit, data on routine management since baseline were documented. Analyses of LDL-C levels and goal achievement were implemented on an LDL-C analysis set, consisting of patients with available LDL-C data at baseline and 1-year follow-up.

The SANTORINI study was performed in accordance with the Declaration of Helsinki and Good Clinical Practice. All participants enrolled into this study provided written informed consent.

### Study objectives

The primary objectives of this analysis of the SANTORINI study were to assess risk-based LDL-C goal attainment (based on the 2019 ESC/EAS dyslipidemia guidelines) (3) and changes in LLT treatment patterns among patients at high and very high CV risk at 1 year, compared with baseline, in France.

### Statistical analysis

All statistical analyses were performed using Statistical Analysis System (SAS®) Version 9.4. Analyses of baseline characteristics, LLTs, LDL-C values and goal achievement were implemented in the LDL-C analysis set. No formal statistical tests were performed. Descriptive statistics are presented as standard summary measures [mean and standard deviation [SD], median and interquartile range [IQR], counts and proportions]. No imputation was performed for missing data. CV risk was calculated using available patient data and applying the 2019 ESC/EAS guidelines CV risk classification (3).

## Results

### Patient population

A total of 9,559 patients were enrolled from the SANTORINI Europe cohort, of whom 9,136 had 1-year follow-up data and were included in the Full Analysis Set (FAS). The FAS-Risk (FAS-R) analysis set consisted of 8,802 patients with available risk classification data, and the LDL-C analysis set consisted of 7,210 patients with available LDL-C data at baseline and 1-year follow-up (Supplementary Figure S1) across 14 countries (Austria [*n* = 246], Belgium [*n* = 328], Denmark [*n* = 211], Finland [*n* = 305], France [*n* = 621], Germany [*n* = 1,543], Ireland [*n* = 74], Italy [*n* = 1,850], Portugal [*n* = 102], Spain [*n* = 956], Sweden [*n* = 163], Switzerland [*n* = 102], the Netherlands [*n* = 329] and United Kingdom [*n* = 380]). Of patients in the LDL-C analysis set, 621 were included in the France cohort and 6,589 were included in the RoE cohort. Baseline patient characteristics and LDL-C levels are presented in Table 1.

**Table 1:** Baseline patient characteristics in France and the RoE^a^.

Characteristic	France (*n* = 621)	RoE (*n* = 6,589)
Male, *n* (%)	453 (73.0)	4,744 (72.0)
Age, years, mean (SD)	65.0 (10.8)	65.0 (10.9)
Hypertension, *n* (%)	356 (57.3)	4,734 (71.9)
Diabetes, *n* (%)	157 (25.3)	2,358 (35.8)
BMI, kg/m^2^, mean (SD)	27.6 (4.7)	28.3 (4.8)
LDL-C, mmol/L, mean (SD)	2.6 (1.3)	2.4 (1.2)
Familial hypercholesterolemia, *n* (%)	53 (8.5)	747 (11.3)
ASCVD, *n* (%)	508 (81.8)	5,013 (76.1)
Smoking history, *n* (%)^b^		
Current	106 (17.1)	1,056 (16.0)
Former	273 (44.0)	2,759 (41.9)
Never	242 (39.0)	2,715 (41.2)
Baseline CV risk classification by investigator^c^		
Very high risk	470 (75.7)	4,703 (71.4)
High risk	151 (24.3)	1,882 (28.6)
Primary care^d^	88 (14.2)	2,542 (38.3)
Secondary care^d^	533 (85.8)	4,963 (75.3)

ASCVD, atherosclerotic cardiovascular disease; BMI, body mass index; LDL-C, low-density lipoprotein cholesterol; RoE, rest of Europe; SD, standard deviation.

a Austria, Belgium, Denmark, Finland, Germany, Ireland, Italy, Portugal, Spain, Sweden, Switzerland, the Netherlands, United Kingdom.

b RoE missing, *n* = 59.

c RoE missing, *n* = 4.

d A patient can be included into primary care and secondary care.

In the France cohort, mean (SD) age was 65.0 (10.8) years, most patients were male (73.0%) and classified as secondary care (85.8%); the mean (SD) LDL-C level at baseline was 2.6 (1.3) mmol/L. Overall, patient characteristics in the France cohort were consistent with those in the RoE cohort (Table 1).

### CV risk classification

In the FAS-R analysis set, investigators classified 20.1% and 79.9% of patients in the France cohort as high and very high CV risk, respectively, compared with 27.3% and 72.7% in the RoE cohort. However, when CV risk was recalculated centrally as per the 2019 ESC/EAS guidelines, 7.1% and 92.9% of patients in the France cohort were considered high and very high risk, respectively, compared with 7.6% and 92.4% in the RoE cohort. CV risk was underestimated in 15.0% of patients in the France cohort and 21.1% of patients in the RoE cohort.

### LLT use at 1-year follow-up

LLT use was assessed and compared between the France cohort and the RoE cohort at 1-year follow-up. Statin monotherapy was the most common regimen used in the France cohort (53.1%), with most patients taking moderate- or high-intensity statins. In the France cohort, 3.4% of patients did not receive LLT. Monotherapy treatment patterns were similar in the RoE cohort (Table 2).

**Table 2:** LLT use at baseline and 1-year follow-up in France and the RoE^a^.

n, %	France (*n* = 621)		RoE (*n* = 6,589)	
	Baseline	1-year follow-up	Baseline	1-year follow-up
No LLT documented	146 (23.5)	21 (3.4)	1,411 (21.4)	196 (3.0)
Total monotherapy	318 (51.2)	363 (58.5)	3,353 (50.9)	3,625 (55.0)
Statin alone^b^	288 (46.4)	330 (53.1)	3,056 (46.4)	3,302 (50.1)
Low intensity	22 (3.5)	23 (3.7)	77 (1.2)	58 (0.9)
Moderate intensity	154 (24.8)	147 (23.7)	1,556 (23.6)	1,534 (23.3)
High intensity	104 (16.8)	155 (25.0)	1,363 (20.7)	1,654 (25.1)
Ezetimibe alone	10 (1.6)	14 (2.3)	128 (1.9)	105 (1.6)
PCSK9i alone	9 (1.5)	10 (1.6)	129 (2.0)	178 (2.7)
Any other oral LLT alone	11 (1.8)	9 (1.5)	40 (0.6)	40 (0.6)
Total combination therapy	157 (25.3)	237 (38.2)	1,825 (27.7)	2,768 (42.0)
Combination statin + ezetimibe^b^	136 (21.9)	196 (31.6)	1,155 (17.5)	1,851 (28.1)
Low-intensity statin	4 (0.6)	4 (0.6)	29 (0.4)	27 (0.4)
Moderate-intensity statin	56 (9.0)	65 (10.5)	393 (6.0)	531 (8.1)
High-intensity statin	69 (11.1)	119 (19.2)	702 (10.7)	1,254 (19.0)
PCSK9i combination	10 (1.6)	30 (4.8)	391 (5.9)	533 (8.1)
Any other oral combination LLT	11 (1.8)	11 (1.8)	279 (4.2)	384 (5.8)

LLT, lipid-lowering therapy; PCSK9i, proprotein convertase subtilisin/kexin type 9 inhibitor; RoE, rest of Europe.

a Austria, Belgium, Denmark, Finland, Germany, Ireland, Italy, Portugal, Spain, Sweden, Switzerland, the Netherlands, United Kingdom.

b Patients with missing statin intensity are included but not detailed.

The proportion of high-CV risk patients receiving statin monotherapy was 61.6% in the France cohort vs. 58.1% in the RoE cohort (low intensity: 7.3% vs. 1.2%; moderate intensity: 35.8% vs. 36.1%; high intensity: 17.2% vs. 19.8%). Other LLT use was similar in the France cohort and RoE cohorts (Supplementary Table S1).

In very high-CV risk patients, statin monotherapy use was 50.4% in France and 46.9% in RoE (low intensity: 2.6% vs. 0.7%; moderate intensity: 19.8% vs. 18.1%; high intensity: 27.5% vs. 27.2%). Other LLT use was comparable in the France cohort and the RoE cohort (Supplementary Table S1).

Monotherapy remained the most frequent form of LLT used at 1-year follow-up and was higher in the France cohort (58.5%) than in the RoE cohort (55.0%). In comparison, total combination therapy use was lower in the France cohort (38.2%) than in the RoE cohort (42.0%; Table 2).

Over the course of 1 year of follow-up, intensification of statin therapy was observed in both cohorts (Table 2). Notably, in the France cohort, the proportion of patients taking no LLT decreased from 23.5% at baseline to 3.4% at 1-year follow-up. Use of high-intensity statin monotherapy and statin plus ezetimibe combination therapy increased from baseline to 1-year follow-up, and a similar pattern was observed in the RoE cohort. On the contrary, the use of low- and moderate-intensity statins, either alone or in combination, remained consistent over time in the France and RoE cohorts (Table 2).

### LDL-C levels and goal attainment at 1-year follow-up

**Figure 1:**
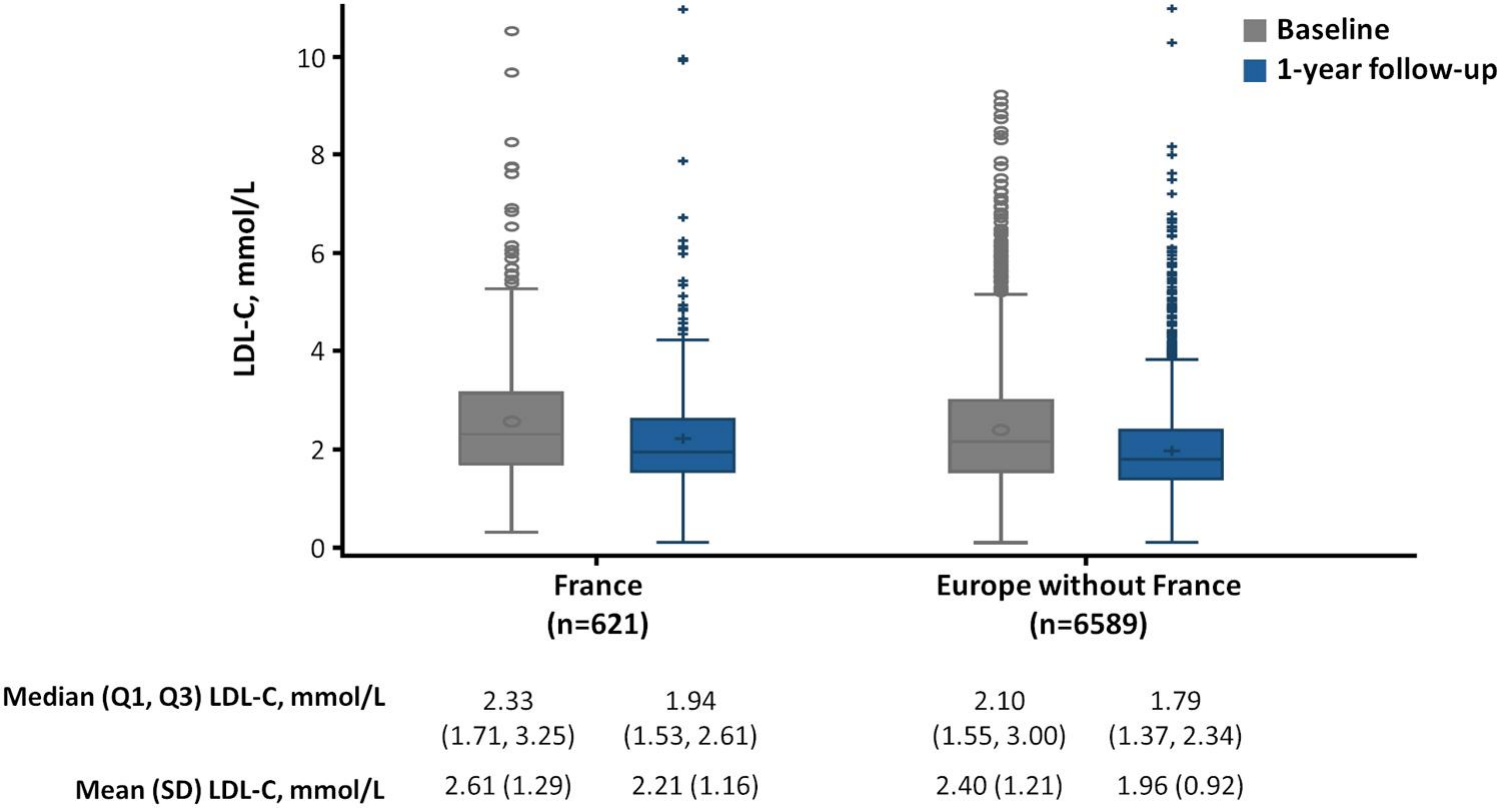
Box plot of LDL-C levels at baseline and 1-year follow-up in France and the RoE^a^. LDL-C, low-density lipoprotein cholesterol; Q1, first quartile; Q3, third quartile; RoE, rest of Europe; SD, standard deviation. ^a^Austria, Belgium, Denmark, Finland, Germany, Ireland, Italy, Portugal, Spain, Sweden, Switzerland, The Netherlands, United Kingdom.

**Figure 2:**
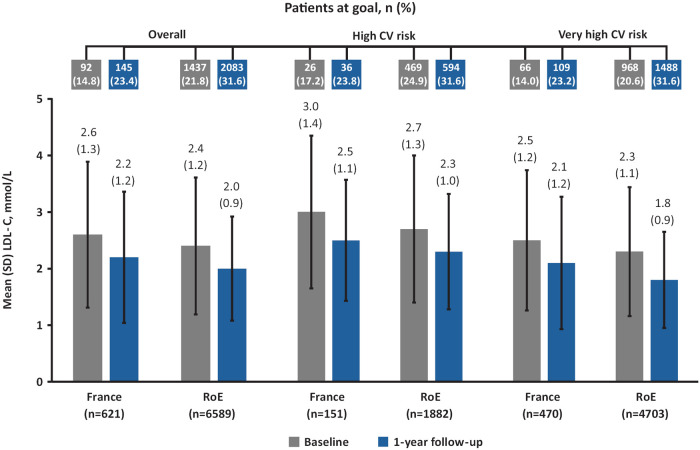
Mean LDL-C levels and proportion of patients at LDL-C goal at baseline and 1-year follow-up in France and the RoE^a^. CV, cardiovascular; LDL-C, low-density lipoprotein cholesterol; RoE, rest of Europe; SD, standard deviation. ^a^Austria, Belgium, Denmark, Finland, Germany, Ireland, Italy, Portugal, Spain, Sweden, Switzerland, The Netherlands, United Kingdom.

Overall, mean (SD) LDL-C level was higher at 1-year follow-up in the France cohort than in the RoE cohort (2.2 [1.2] vs. 2.0 [0.9] mmol/L; Figures 1, 2). Fewer patients achieved their risk-based LDL-C goals in the France cohort (23.4%) compared with the RoE cohort (31.6%; Figure 2).

In the high- and very high-CV risk groups, mean (SD) LDL-C levels were higher at 1-year follow-up in the France cohort compared with the RoE cohort. Furthermore, LDL-C goal attainment was lower for both CV risk groups in the France cohort compared with the RoE cohort (Figure 2, Supplementary Figure S2).

## Discussion

Overall, in the France cohort of the SANTORINI study, the majority of patients at elevated CV risk were older, male, and in secondary care; the mean (SD) LDL-C level at 1-year follow-up was 2.2 (1.2) mmol/L. In addition, CV risk was underestimated in approximately 1 in 7 patients by the investigators. After a centrally reassessed CV risk calculation, approximately 93% of patients were categorized as very high CV risk. Such findings indicate that further work is needed to ensure the 2019 ESC/EAS guidelines are implemented in clinical practice so that patients are treated accurately according to their CV risk (3).

Further, we observed improvements in mean LDL-C levels of 0.4 mmol/L and in LDL-C guideline-recommended goal attainment in both high- and very high-CV risk patients over 1-year of longitudinal follow-up. These findings were likely driven by an initiation of LLT in patients not taking any LLT at baseline and an increase in the use of combination therapies, which followed a trend consistent with what was observed in the RoE cohort.

However, mean LDL-C levels were higher in the France cohort than in the RoE cohort at 1-year follow-up, as French patients had less LDL-C control than patients in the RoE. The mean LDL-C levels for high- and very high-CV risk patients were approximately 0.8 mmol/L above respective 2019 ESC/EAS guideline risk-based LDL-C goals. This might be associated with the significant proportion of patients in the France cohort only receiving monotherapy at 1-year follow-up. For example, the proportion of patients receiving high-intensity statin doses at 1-year follow-up was 25.0%, which may account for the low LDL-C goal attainment, despite improvements in LDL-C levels throughout Europe at 1-year follow-up (9).

Combination therapy was the most common approach for treatment intensification over the follow-up period. However, in the France cohort, a lower proportion of patients received combination therapy compared with the RoE cohort. This may highlight a lack of LLT intensification in clinical practice because higher proportion of patients were in secondary care in the France cohort, in which greater use of more intensive LLTs would be expected. For instance, a comparative study of DYSIS and DYSIS II found that while there were substantial improvements in LDL-C goal attainment, the majority of patients in France were prescribed higher doses of statins and the use of combination therapies did not change (10). In addition, a recent nationwide study using real-world data from the French National Health Data System found that despite favorable reimbursement for statins and ezetimibe combination therapy in patients with ASCVD who had LDL-C levels >70 mg/dL, only 13% of patients with ASCVD received statin plus ezetimibe combination therapy, and fewer than 0.5% received a proprotein convertase subtilisin/kexin type 9 inhibitor (11). These findings align with our observation of suboptimal LDL-C goal attainment in France and highlight the need for better implementation strategies. These could include further education for physicians in France regarding the ESC/EAS guidelines, which could increase awareness of combination therapies and their benefits in LDL-C level reduction. Promoting earlier use of combination therapies may also help high- and very high-CV risk patients to achieve LDL-C goals.

Contributory factors to approximately three-quarters of patients in the France cohort failing to achieve the 2019 ESC/EAS guideline LDL-C goals at 1-year follow-up may include underutilization of combination therapies and CV risk underestimation. In addition, treatment guidelines recommend a stepwise approach—beginning with statin monotherapy at the highest tolerated dose (3). However, statins alone are often insufficient in helping patients to achieve their LDL-C goals, and there is a need for additional LLT intensification in France. For example, a recent French simulation analysis highlighted the value of combination therapy use in increasing LDL-C goal attainment in statin-intolerant patients who were not at goal with low-intensity statin or ezetimibe monotherapy. In these patients, it was shown that stepwise treatment optimization with ezetimibe and bempedoic acid could increase the proportion at LDL-C goal from 0% to approximately 40% (12). This finding supports the use of combination therapy and the introduction of new LLTs (13–16).

Previously, access to additional therapies was limited in France and prevented physicians from intensifying treatment beyond statins and ezetimibe. However, the recent availability and reimbursement of bempedoic acid in France may close the gap between guideline-recommended treatment intensification strategies and clinical practice. Successful uptake will depend on physician education and awareness, as well the promotion of early combination therapy use in clinical practice.

Inherent within this small-sized observational study are several study design-related limitations. As the study is observational in nature, no formal causal inference can be made between treatment intake and LDL-C patterns. Furthermore, this was a descriptive analysis with no formal hypotheses tested; therefore, caution should be used when interpreting any associations. Another potential limitation of this study is the representativeness of both the France and RoE cohorts due to patient loss between baseline and the LDL-C analysis set in each country. The findings of this analysis may not be generalizable to routine clinical practice if these cohorts do not reflect real-world patient populations. Country-specific differences in healthcare systems may result in variations in access to drugs, initial prescribers, prescription renewal practices, reimbursement policies, and frequency of patient visits across countries could influence treatment patterns and patient outcomes due to selection bias. For instance, in France it is prohibited to prescribe ezetimibe in combination with a statin for a first prescription of LLT, even for secondary prevention. This severely limits LDL-C goal attainment because it delays the initiation of combination therapy by at least 3 months after the onset of acute coronary syndrome. Thus, owing to system-level factors, direct comparisons between France and the RoE may have limited validity and impact the generalizability across regions. There may also be selection bias due to differences in the accuracy of investigator follow-up. As such, the France cohort in this study may not be fully representative of real-world lipid management in France.

## Conclusions

The findings from the France cohort of the SANTORINI study were consistent with those observed in the RoE cohort. CV risk was underestimated in 15.0% of patients in the France cohort, and only approximately one-quarter of patients reached their LDL-C goals at 1-year follow-up. Thus, most patients remained at an increased risk of CV events. These results highlight the need for further strategies to increase the use of combination therapies. Healthcare professionals should receive improved guideline-related training, and patients should have increased access to more intensive treatments to optimize lipid management and reduce CV risk.

## Data Availability

De-identified individual participant data and applicable supporting clinical study documents are available on request, depending on circumstances, at https://vivli.org. In cases in which clinical study data and supporting documents are provided pursuant to the sponsor's policies and procedures, the sponsor will continue to protect the privacy of the clinical study participants. Details on data sharing criteria and the procedure for requesting access can be found at https://vivli.org/ourmember/daiichi-sankyo/.

## Author's note

A list of the SANTORINI study investigators is provided in Supplementary Table S2 and Supplementary Table S3.
