# Supplementary material for: Lipid management in high cardiovascular risk patients in France: a comparison with rest of Europe patients from 1-year follow-up of SANTORINI
Source: Front Cardiovasc Med. 2026 Mar 12;13:1748457. doi: 10.3389/fcvm.2026.1748457 (PMC13017259; doi:10.3389/fcvm.2026.1748457)
Supplement: Supplementary file 1 [file Datasheet1.docx]

# Supplementary material

##
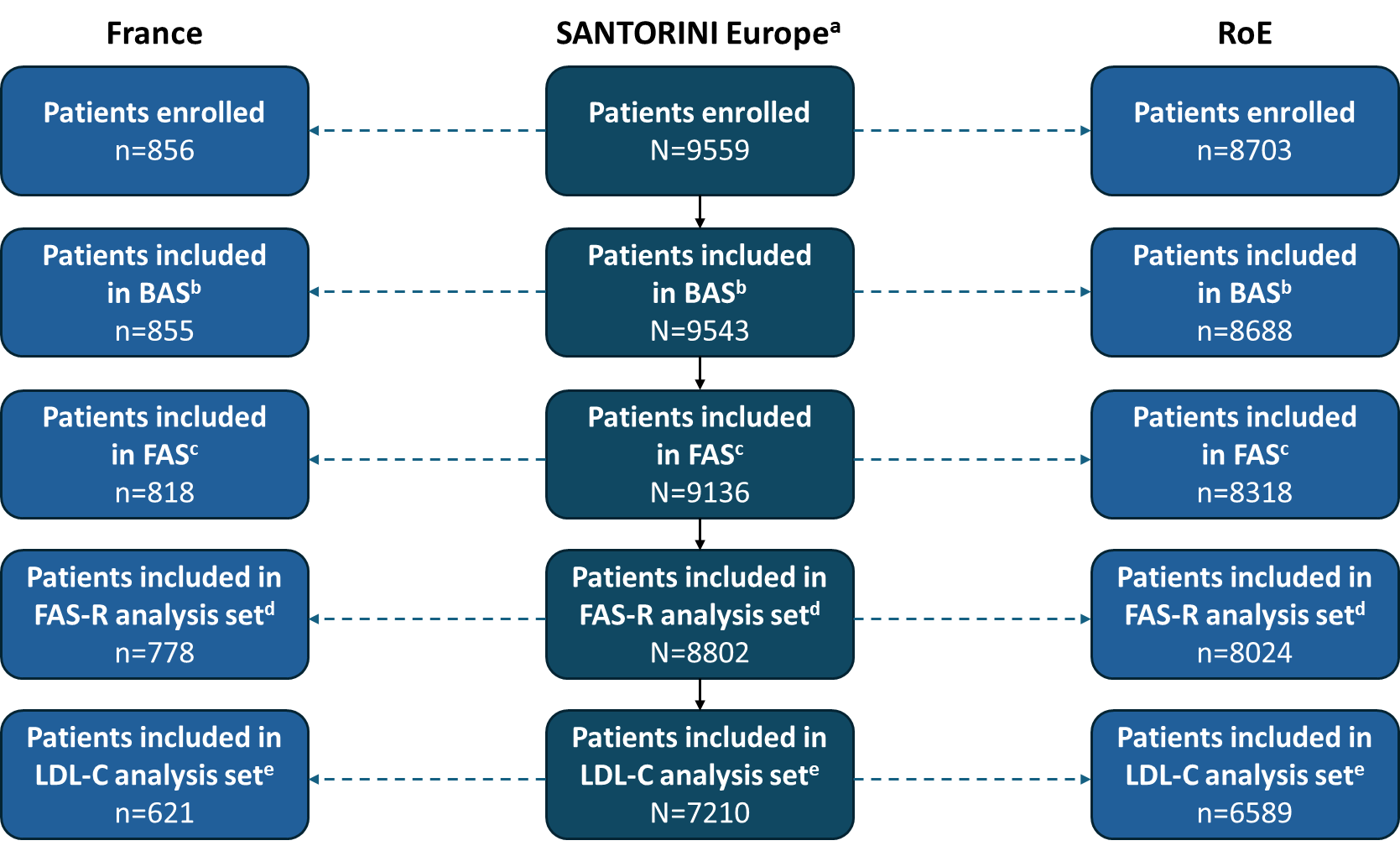
FIGURE S1 Patient flowchart. BAS, Baseline Analysis Set; FAS, Full Analysis Set; FAS-R, Full Analysis Set for Risk; LDL-C, low-density lipoprotein cholesterol; LLT, lipid-lowering therapy; RoE, rest of Europe. ^a^Austria, Belgium, Denmark, Finland, France, Germany, Ireland, Italy, Portugal, Spain, Sweden, Switzerland, the Netherlands, United Kingdom. ^b^Including all patients with electronic case report form documentation available and adequate baseline information (age, sex, baseline LLT use, and LDL-C level). ^c^Including all patients from the BAS with available 1-year follow-up visit data (at least visit date is required). ^d^Excluding patients with missing risk classification. ^e^Including patients who had LDL-C data at both the baseline visit and 1-year follow-up visit.

## FIGURE S2 Proportion of patients at LDL-C goal at baseline and 1-year follow-up in France and the RoE compared with guideline-recommended goals. CV, cardiovascular; EAS, European Atherosclerosis Society; ESC, European Society of Cardiology; LDL-C, low-density lipoprotein cholesterol; RoE, rest of Europe; SD, standard deviation. ^a^Based on the 2019 ESC/EAS dyslipidemia guidelines. ^b^Austria, Belgium, Denmark, Finland, Germany, Ireland, Italy, Portugal, Spain, Sweden, Switzerland, the Netherlands, United Kingdom.

##
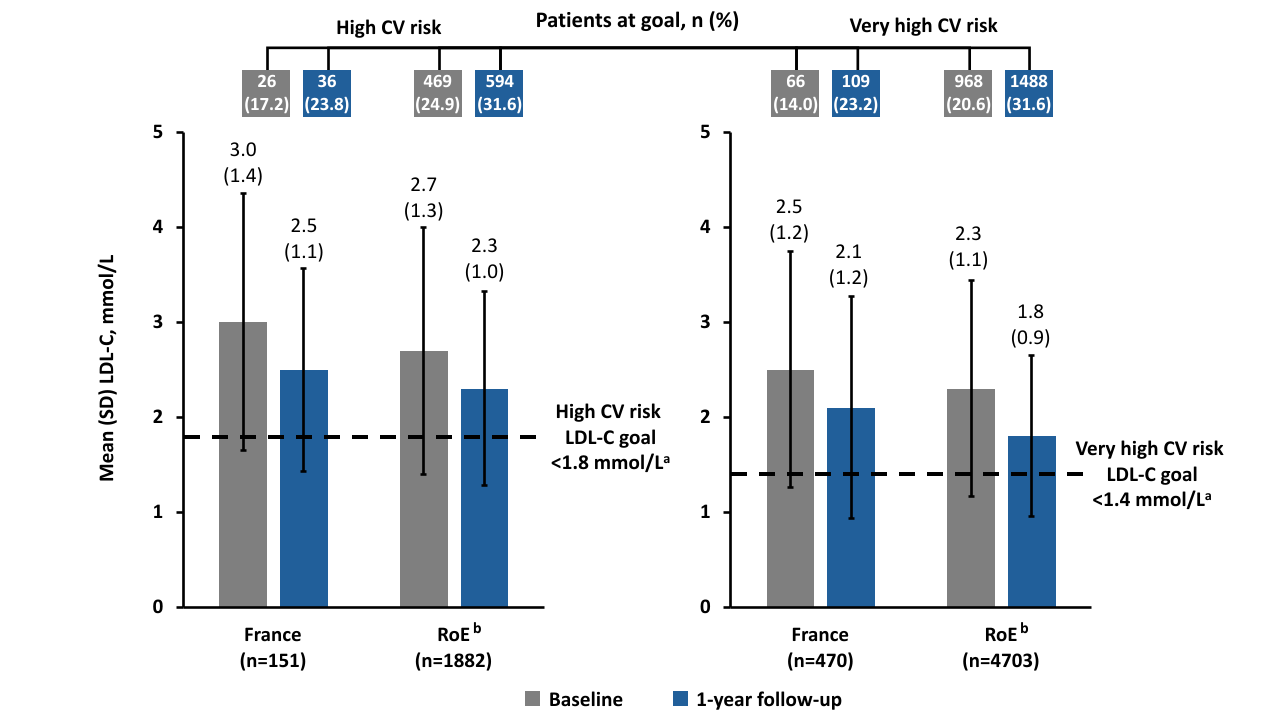


## TABLE S1 LLT use stratified by CV risk classification in France and the RoE^a^ at 1-year follow-up.

| **n, %** | **France**  **(n=621)** | | | **RoE (n=6589)** | | |
| --- | --- | --- | --- | --- | --- | --- |
|  | **High risk**  **(n=151)** | | **Very high risk**  **(n=470)** | **High risk**  **(n=1882)** | **Very high risk**  **(n=4703)** | |
| **No LLT documented** | 8 (5.3) | 13 (2.8) | | 97 (5.2) | | 98 (2.1) |
| **Total monotherapy** | 109 (72.2) | 254 (54.0) | | 1185 (63.0) | | 2437 (51.8) |
| Statin alone^b^ | 93 (61.6) | 237 (50.4) | | 1093 (58.1) | | 2206 (46.9) |
| Low intensity | 11 (7.3) | 12 (2.6) | | 23 (1.2) | | 35 (0.7) |
| Moderate intensity | 54 (35.8) | 93 (19.8) | | 679 (36.1) | | 853 (18.1) |
| High intensity | 26 (17.2) | 129 (27.5) | | 373 (19.8) | | 1280 (27.2) |
| Ezetimibe alone | 7 (4.6) | 7 (1.5) | | 39 (2.1) | | 66 (1.4) |
| PCSK9i alone | 3 (2.0) | 7 (1.5) | | 37 (2.0) | | 141 (3.0) |
| Any other oral LLT alone | 6 (4.0) | 3 (0.6) | | 16 (0.9) | | 24 (0.5) |
| **Total combination therapy** | 34 (22.5) | 203 (43.2) | | 600 (31.9) | | 2168 (46.1) |
| Combination statin + ezetimibe^b^ | 29 (19.2) | 167 (35.5) | | 362 (19.2) | | 1489 (31.7) |
| Low-intensity statin | 0 | 4 (0.9) | | 7 (0.4) | | 20 (0.4) |
| Moderate-intensity statin | 13 (8.6) | 52 (11.1) | | 144 (7.7) | | 387 (8.2) |
| High-intensity statin | 15 (9.9) | 104 (22.1) | | 202 (10.7) | | 1052 (22.4) |
| PCSK9i combination | 3 (2.0) | 27 (5.7) | | 135 (7.2) | | 398 (8.5) |
| Any other oral combination LLT | 2 (1.3) | 9 (1.9) | | 103 (5.5) | | 281 (6.0) |

CV, cardiovascular; LLT, lipid-lowering therapy; PCSK9i, proprotein convertase subtilisin/kexin type 9 inhibitor; RoE, rest of Europe.

^a^Austria, Belgium, Denmark, Finland, Germany, Ireland, Italy, Portugal, Spain, Sweden, Switzerland, the Netherlands, United Kingdom. ^b^Patients with missing statin intensity are included but not detailed.

**TABLE S2 SANTORINI Steering Committee**

| **Member** | **Affiliation** |
| --- | --- |
| Kausik K. Ray, MD, MPhil (Co-Chair) | Imperial College London, London, UK |
| Alberico L. Catapano, MDhc, PhD (Co-Chair) | University of Milan and Multimedica IRCCS, Milan, Italy |
| Carlos Aguiar, MD | Heart Institute, Carnaxide, Portugal |
| Marcello Arca, MD | Sapienza Università di Roma, Rome, Italy |
| Derek L. Connolly, MBChB, PhD | Sandwell and West Birmingham NHS Trust, Birmingham City Hospital; University of Birmingham, Birmingham, UK |
| Mats Eriksson, MD, PhD | Karolinska University Hospital, Stockholm, Sweden |
| Jean Ferrières, MD, PhD | Toulouse Rangueil University Hospital, Toulouse University School of Medicine, Toulouse, France |
| Per Hildebrandt, MD, DMSc | Frederiksberg Heart Clinic, Frederiksberg, Denmark |
| Ulrich Laufs, MD | University Hospital Leipzig, Leipzig, Germany |
| Jose M. Mostaza, MD, PhD | La Paz-Carlos III Hospital, Madrid, Spain |
| David Nanchen, MD, MSc | University of Lausanne, Lausanne, Switzerland |
| Ernst Rietzschel, MD, PhD | Ghent University and Ghent University Hospital, Ghent, Belgium |
| Timo Strandberg, MD, PhD | University of Helsinki and Helsinki University Hospital, Helsinki, Finland, and University of Oulu, Oulu, Finland |
| Hermann Toplak, MD | Medical University of Graz, Graz, Austria |
| Frank L. J. Visseren, MD, PhD | University Medical Center Utrecht, Utrecht, The Netherlands |

**TABLE S3 SANTORINI Investigators and study sites**

| **Investigator Name** | **Institution** |
| --- | --- |
| **Austria** |  |
| Univ. Prof. Dr. med. univ. Herrmann Toplak | Medizinische Universität Graz |
| Univ.-Prof. Dr. Christian Hengstenberg | Allgemeines Krankenhaus der Stadt Wien |
| Prim. Univ. Prof. Dr. Martin Clodi | Konventhospital der Barmherzigen Brüder Linz |
| Univ. Prof. Dr. Bernhard Ludvik | Krankenhaus Rudolfstiftung |
| Ao. Univ.-Prof. Dr. Christoph Ebenbichler | Medizinische Universität Innsbruck |
| Ao. Univ. Prof. Dr. Gerit-Holger Schernthaner | Allgemeines Krankenhaus der Stadt Wien |
| Dr. Ursula Hanusch | Zentrum für Klinische Studien Dr. Hanusch GmbH |
| Prim. Univ. Prof. Dr. Johannes Auer | Krankenhaus St. Josef Braunau GmbH |
| Priv. Doz. Dr. Andreas Schober | Krankenhaus Nord – Klinik Floridsdorf |
| PD Dr. Hannes Alber | KABEG Klinikum Klagenfurt am Wörthersee |
| Univ. Prof. Dr. Andreas Zirlik | LKH-Univ. Klinikum Graz |
| Dr. med. univ. Evelyn Fließer-Görzer | Ordination Dr. med. univ. Evelyn Fließer-Görzer |
| Prim. Dr. Thomas Maca | Ordination Dr. Thomas Maca / Evangelisches KH Wien |
| Dr. Reinhard Sock | Ordination Dr. Reinhard Sock |
| Ao.Univ. Prof. Dr. Thomas Stulnig | Krankenhaus Hietzing mit neurologischem Zentrum Rosenhügel |
| Prim. Univ.- Prof. Dr. Raimund Weitgasser | Privatklinik Wehrle-Diakonissen |
| Univ. Doz. Dr. Alexander Kober | Ordination Universitätsdozent Dr. Alexander Kober |
| Dr. med. Carmen Gelsinger | Internistische Facharztpraxis Frau Dr. Carmen Gelsinger |
| **Belgium** |  |
| Professeur Fabian DEMEURE | CENTRE HOSPITALIER UNIVERSITAIRE UCL NAMUR |
| Docteur Yvan CALOZET | CABINET DE MEDECINE GENERALE |
| Docteur FRANK COOLS | AZ KLINA |
| Dr. TOM SARENS | AZ SINT BLASIUS |
| Dr. PETER VERSYCK | Huisartsenpraktijk Dr. Versyck |
| Docteur FILIP VANSTECHELMAN | AZ SINT ELISABETH ZOTTEGEM |
| Dr. LUC CAPIAU | Huisartsenpraktijk BVBA Dr. Capiau Luc |
| Dr. KARL DUJARDIN | Az delta |
| Dr. PHILIPPE VANDUYNHOVEN | Asz |
| Dr. ALEX HEYSE | Az glorieux |
| Docteur LUC JANSSENS | Imeldaziekemhuis |
| Docteur Filip DE MAN | Ciniques de l'europe |
| Dr. IVAN ELEGEERT | Az groeninge |
| Dr. RAF ROELANDT | Jan yperman ziekenhuis |
| Docteur Philippe EVRARD | Chc montlegia |
| Docteur Rachid MAAMAR | Centre hospitalier regional de huy |
| Docteur Philippe VAN DE BORNE | Cub hopital erasme |
| Docteur HANS VANDEKERCKHOVE | Az sint lucas & volkskliniek |
| Docteur EVA DECALUWE | Sint trudo |
| Docteur BERNARD COSYNS | Uz brussel |
| Docteur Olivier DESCAMPS | Centre hospitalier jolimont-lobbes |
| Docteur Denis PIETERS | Clinique saint luc |
| Docteur GEERT HOLLANDERS | Private practice |
| Docteur Emmanuel CATEZ | Chu brugmann - site victor horta |
| Dr. AXEL DE WOLF | Privé praktijk cardiologie dr. De wolf |
| Prof. Dr. Ernst RIETZSCHEL | Universitair ziekenhuis gent |
| Docteur Panagiotis XAPLANTERIS | Chu saint pierre |
| **Denmark** |  |
| Dr. Per Rossen Hildebrandt | Frederiksberg Hjerteklinik |
| MD, Head of research Morten Kofod Lindhardt | Holbaek Sygehus |
| Consultant, associate professor Helle Klingenberg Iversen | Stroke center Rigshospitalet |
| Professor Gunnar Gislason | Gentofte Hospital |
| Anders Ørsted Schultz | Sygehus Sønderjylland |
| Jens Lomholdt | Slagelse Sygehus |
| Hanne Krarup Christensen | Bispebjerg-Frederiksberg Hospital |
| Kristian Thomsen | Sydvestjysk Sygehus ESbjerg |
| Merete Heitmann | Bispebjerg/ Frederiksberg Hospital |
| Jens Broennum-Schou | Amager Hospital |
| Soren Galatius | Hjerteklinik Østerbro |
| Christian Tuxen | Frederiksberg Hjerteklinik |
| **Finland** |  |
| Professor Timo Strandberg | Geri-Med Oy |
| MD Sakari Sulosaari | Aava Kerava Medical Center |
| MD Satu Näppi | Terveystalo Tampere |
| MD Kai Nyman | StudyCor Oy |
| MD Marta Abraham | Terveystalo Kalajoki |
| MD Ilkka Kantola | Turku University Hospital |
| MD Sakari Kekki | Pihlajalinna Ite Joensuu |
| MD Kristiina Averson | Terveystalo |
| Professor Janne Hukkanen | Oulu University Hospital |
| MD Pirkko Korsoff | Satasairaala |
| MD Annukka Lagerstedt | Terveystalo Kokkola |
| MD Jyrki Taurio | Tampere Univestity hospital |
| MD Nelli Valkama | Sastamalan seudun sosiaali- ja terveyspalvelut |
| MD Juha Hartikainen | Mehiläinen Kuopio |
| MD Veli-Matti Häggman | Terveystalo Myyrmäki |
| MD Sari Risku | Seinäjoki Central Hospital |
| MD Mikko Syvänne | Terveystalo Myyrmäki |
| MD Robert Paul | Mehiläinen Turku Kauppiaskatu |
| MD Harri Hietanen | Pihlajalinna Dextra |
| MD Arto Strandberg | Finla Työterveys Vantaa |
| MD Jussi Talka | Lappeenrannan Työterveys |
| MD Tommi Aaltokallio | Lappeenrannan Työterveys |
| **France** |  |
| Docteur Nicolas DELARCHE | Centre Hospitalier de Pau |
| Docteur Maxime FAYARD | Centre Hospitalier de Chalon sur Soane |
| Docteur Serge COHEN | Cabinet Médical |
| Professeur Emile FERRARI | Hôpital Pasteur |
| Docteur Fahmi GHANEM | Cabinet Médical du Docteur GHANEM |
| Docteur Gérard DASSA | Cabinet médical |
| Doctor JULIEN LENESTOUR | Groupe hospitalier rochelle ré-aunis |
| Docteur Jean-Noël LABEQUE | Centre de cardiologie et d'exploration de la cote basque |
| Docteur Eric DECOULX | Hôpital Guy Chatillez |
| Docteur Benjamin CAZE | Cabinet Médical |
| Docteur Gilles LEMESLE | Institut Coeur Poumons |
| Docteur Laurent DELORME | Clinique du Pont de Chaume |
| Docteur Claire BOULETI | Centre Hospitalier Universitaire Poitiers |
| Docteur Jeannot RAZAFY | Cabinet Médical |
| Docteur Olivier AZZANO | Cabinet médical Le Loredan |
| Professeur Gilles BARONE ROCHETTE | Centre Hospitalier Grenoble Alpes |
| Docteur Jean-Michel TARTIERE | Hôpital Sainte Musse |
| Docteur Gilles MONTALESCOT | Hôpital de la Pitié Salpétrière |
| Docteur Guillaume CAYLA | Centre Hospitalier Carémeau |
| Docteur Jacques BERLAND | Clinique Saint Hilaire |
| Dr Sébastien CAUDMONT | Cabinet Médical Louis Blanc |
| Docteur Renaud SOLANET | Cabinet Médical du Docteur SOLANET |
| Docteur Franck BOCCARA | Hôpital Saint Antoine |
| Jean-Noël ANDARELLI | Hôpital Marie Lannelongue |
| Docteur Gilles LEVY | Clinique du Millénaire |
| Docteur Vincent TROUSSARD | Centre Hospitalier de Lisieux |
| Docteur Stéphane MINGAM | Maison de santé Mont Gaillard |
| Docteur François POULAIN | Cabinet de Cardiologie |
| Docteur Vincent PROBST | Centre Hospitalier Nantes |
| Professeur Alain FURBER | Centre Hospitalier d'Angers |
| Professeur Bertrand CARIOU | Hopital guillaume et rene laennec |
| Docteur Grégoire RANGE | Hopital louis pasteur |
| Docteur Martin AUDONNET | Hôpital arcachon |
| Docteur Jérôme CLERC | Centre Hospitalier de Compiègne |
| Docteur Thierry LEMERCIER | Cabinet Médical du Docteur LEMERCIER |
| Docteur Jean-Philippe MOURET | Centre hospitalier la timone |
| Docteur DOCTEUR RACHENNE | Clinique d'argonay |
| Docteur Pierre-François LESAULT | Hôpital privé de l'Estuaire |
| Docteur Boutheina ZEGHIDI | Clinique de Meudon |
| Professeur Jean FERRIERES | Centre hospitalier universitaire de toulouse |
| Docteur Damien BROUCQSAULT | Centre hospitalier arras |
| Docteur Christian DUROY | Cabinet médical Dr DUROY |
| Docteur Victor ABOYANS | Centre Hospitalier universitaire de Limoges |
| Docteur Dominique STEPHAN | Nouvel Hôpital Civil de Strasbourg |
| **Germany** |  |
| Prof. Dr. med. Ulrich Laufs | Universitätsklinikum Leipzig |
| Prof. Dr. Gregor Simonis | Zentrum für klinische Prüfungen in der Facharztzentrum Dresden-Neustadt GbR |
| Dr. Frank Schaper | Studienzentrum Dr. Faulmann |
| Dr. Ulrike Schatz | Universitätsklinikum Carl Gustav Carus |
| Dr. Hans-Holger Ebert | Gemeinschaftspraxis Herz Riesa |
| Dr. Christoph Axthelm | Hausärztlich-Kardiologisches MVZ Am Felsenkeller GmbH |
| Dr. Wolfram Oettler | Praxis für Gefäßmedizin |
| Dr. Matthias Weißbrodt | Kardiologische Gemeinschaftspraxis |
| Dr. Eigk Grebe | MediClin MVZ Leipzig |
| Dr. Ina Wittig | Praxis für Gefäßerkrankungen und Gerinnungsstörungen Leipzig |
| Dr. Antje Spens | MVZ Stoffwechselmedizin Leipzig |
| Dr. Jens Taggeselle | Kardiologische Praxis |
| Dr. Ulrike Spengler | Kardiologische Praxis |
| MUDr. Andreas Hagenow | Kardiologische Praxis |
| Prof. Dr. Axel Schlitt | Paracelsus-Harz-Klinik Bad Suderode |
| Dr. med. Frank Menzel | Praxis Dr. Menzel |
| Dr. Jörg Langel | Kardiologische Praxis |
| PD Dr. Jens Gerth | Heinrich-Braun-Klinikum gGmbH |
| Dr. Alexander Leißring | MVZ Aue |
| Dr. Dieter Enders | Kardiologische Praxis |
| Dr. Heiko Stellmach | Kardiologische Praxis |
| Dr. Ursula Kassner | Charité Campus Virchow |
| Dr. Werner A. Rieker | SKP Studienzentrum der kardiologischen Praxisgemeinschaft Rankestrasse GmbH |
| Dr. Claudia Zemmrich | MVZ Dres. Ramdohr - Praxis für Cardiovascular und Ultraschalldiagnostik |
| Dr. Matthias Claus | Dr. Christine Möller und Dr. Matthias Claus GbR |
| Dr. Anne Winkelmann | Kardiologische Facharztpraxis Dr. Anne Winkelmann |
| Prof. Dr. med. Steffen Behrens | Vivantes Humboldt-Klinikum |
| Dr. Thomas Vogtmann | Kardiologische Gemeinschaftspraxis Potsdam |
| Dr. Ulrich Wolf | Kardiologische Praxis |
| Prof. Dr. med. Oliver Ritter | Städtisches Klinikum Brandenburg |
| Dr. Michael Rother | Praxis Dr. Michael Rother |
| Prof. Hüseyin Ince | Universitätsmedizin Rostock |
| Dr. Heinrich Prophet | Nephrocare Rostock GmbH - Lipidambulanz Praxis Rostock-Südstadt |
| Dr. Behrus Subin | CardioMed an der Alster |
| Dr. med. Christina Paitazoglou | Cardiologicum Hamburg-Wandsbek |
| Dr. Thomas Twisselmann | Kardiologie am Tibarg |
| Prof. Dr. Joachim Schofer | MVZ Prof. Detlef Mathey, Prof. Schofer GmbH |
| Dr. Andreas Wilke | Kardiologische Praxis Papenburg |
| Dr. Detelin Denchev | Kardiologische Praxis Papenburg |
| Dr. Muhammed Gercek | Herz - u. Diabeteszentrum NRW |
| Dr. Uwe Gerbaulet | Gemeinschaftspraxis Dr. Jens Biesenbaum und Dr. Uwe Gerbaulet |
| Prof. Dr. med. Stephan Gielen | Klinikum Lippe GmbH |
| Prof. Dr. Andreas Götte | St. Vincenz-Krankenhaus GmbH |
| Dr. Karl-Friedrich Appel | B. Braun Ambulantes Herzzentrum MVZ GmbH |
| Dr. med. Jörg Simon | MVZ Altstadt-Carree Fulda GmbH |
| Dr. Katja Gollisch | Universitätsmedizin Göttingen |
| Dr. med. Markus Baar | Kardiologische Praxis Northeim |
| Dr. med. Matthias Stratmann | Kardiologische Gemeinschaftspraxis |
| Dr. med. Nils Dyckmanns | Sankt-Johannes-Hospital |
| Dr. med. Thomas Wetzel | Kardiologische Gemeinschaftspraxis Dres. Wetzel/Willgeroth |
| Dr. Bernd Schütz | Dres. Erdmann/Schütz/Sicken |
| Dr. Axel Schaefer | Medizentrum Essen Borbeck |
| Dr. Afif Haj-Yehia | Kardiologie-Praxis |
| Dr. Ludger Rose | Zentrum für Diabetes und Gefäßerkrankungen Münster |
| Dr. Christian Fechtrup | Praxis für Innere Medizin |
| Dr. Katrin Gebauer | Universitätsklinikum Münster |
| Dr. med. Sven Meyer | St. Josefs-Hospital Cloppenburg |
| Dr. Martin Klutmann | Kardiologische Praxis Aachen |
| Dr. Astrid Schmidt-Reinwald | Diabetologische Schwerpunktpraxis |
| Prof. Dr. Nikos Werner | Krankenhaus der Barmherzigen Brüder Trier |
| Prof. Dr. Rainer Zotz | Marienhaus-Klinikum Eifel Bitburg |
| Prof. Dr. Christine Espinola-Klein | Universitätsklinikum Mainz |
| Prof. Dr. med. Philipp Wild | Universitätsklinikum Mainz |
| Prof. Dr. Sabine Genth-Zotz | St.Vincenz - u. Elisabeth-Hospital |
| Dr. med. Fabian Krämer | Zentrum für Prävention und Rehabilitation |
| Dr. Thomas Horacek | Forschungszentrum-Ruhr / KliFoCenter GmbH |
| Prof. Veselin Mitrovic | Kerckhoff-Klinik GmbH |
| Dr. Wolfgang Jungmair | Kardiologische Praxis |
| Dr. Jörg Hintze | Gemeinschaftspraxis Dres. J. Hintze und M. Grundner |
| Prof. Dr. Stephan Steiner | St. Vincenz-Krankenhaus |
| Dr. Christoph Kadel | Klinikum Frankfurt Hoechst GmbH |
| Dr. Jennifer Wolf | SHG Klinik Völklingen |
| Dr. Josef Lißmann | Praxis |
| Prof. Dr. med. Michael Böhm | Universitätsklinikum des Saarlandes |
| Dr. Bernd Hammer | Kardiologische Praxis |
| Dr. Karl Bruck | Kardiologische Praxis |
| Prof. Dr. Tim Süselbeck | Kardiologische Praxisklinik Ludwigshafen |
| Dr. Holger Killat | Kardiologische Praxis |
| Prof. Dr. Dr. Stephan Schirmer | Kardiopraxis Schirmer |
| Dr. Peter Salbach | ze:ro Praxen Mannheim |
| Dr. Ksenija Stach | Universitätsklinikum Mannheim |
| Prof. Christian Erbel | Uniklinikum Heidelberg |
| Prof. Grigorios Korosoglou | GRN Klinik Weinheim |
| Dr. med. Oliver Scheuermann | Praxis für Innere Medizin / Kardiologie - Dr. med. E. Persicke / Dr. med. O. Scheuermann |
| PD Dr. med. Ralph Bosch | Cardio Centrum Ludwigsburg-Bietigheim |
| Prof. Dr. med. Erwin Blessing | SRH Klinikum Karlsbad-Langensteinbach |
| Prof. Dr. med. Stefan Hardt | Kardiologie im Friedrichspalast Bruchsal |
| Dr. med. Stefan Leggewie | Universitätsklinikum Freiburg Universitäts-Herzzentrum |
| Dr. med. Michael Schmid | KARDIO-IN Kardiologische Gemeinschaftspraxis Ingolstadt |
| PD Dr. med. Heiko Methe | Kliniken an der Paar - Krankenhaus Aichach |
| Prof. Dr. med. Harry W. Hahmann | Kardiologie Oberschwaben – Bodensee Herz- und Gefäßpraxis |
| Dr. med. Norbert Jahnke | Studienzentrum der Herzklinik Ulm GbR |
| Dr. med. Alexander Stadelmann | Kardiologie am Weißen Turm |
| Prof. Dr. med. Michael Jeserich | Gemeinschaftspraxis Drs. Haggenmiller/Jeserich Kardiologie-Angiologie-Innere Medizin |
| Dr. med. Johannes Haas | Gemeinschaftspraxis für Herz und Lunge |
| Dr. med. Steffen Schnupp | Klinikum Coburg GmbH |
| Dipl. med. Petra Herrmann | Praxis Dipl. - Med. Petra Herrmann - Fachärztin für Innere Medizin |
| Prof. Dr. Martin Fassnacht-Capeller | Universitätsklinikum Würzburg Medizinischen Klinik und Poliklinik I |
| Dipl.med. Karl-Heinz Schermaul | Praxis Dipl.-med. Schermaul |
| Dr. med. Ronald Naumann | MVZ Polymed |
| Dr. med. Jan Ernstberger | MVZ am Küchwald |
| Marlena Retkowska | MediClin Reha-Zentrum Spreewald |
| Prof. Dr. Stephan Jacob | Praxis für Prävention und Therapie |
| Dr. med. Frank Hamann | Studienzentrum der Inneren Medizin I & II am Klinikum Konstanz |
| Dr. med. Mahmoud Delonge | Praxis Delonge |
| Sagy El-Meadawy | Downtown Clinic Aachen |
| Dr. med. Katharina Hellhammer | Elisabeth Krankenhaus Essen |
| Dr. Katrin Eberhard | Cardiologicum Dresden/Pirna |
| Prof. Martin Köhrmann | Universitätsklinikum Essen |
| Dr. Nadine Waessnig | Dres. Günther/Kolschmann |
| Dr. Markus Buresch | Drs. Bauer/Buresch/Kösler |
| Prof. Karl-Josef Osterziel | Oberpfalz Research GbR |
| Dr. Eva Olesch | Drs. Knoebel @ Partner |
| Prof. Dr. Jens Jung | Klinikum Worms |
| Dr. Annika Voigt | Kardiologische Praxis |
| Dr. Diethard Predel | Praxis für Innere Medizin und Angiologie |
| Dr. Jörg-Eike Scholle | Evangelisches Krankenhaus Gelsenkirchen |
| Dr. Clemens Schiffer | CC Cleve-Studiencenter |
| Dr. med. Stefan Lukannek | Kardiologische Praxis Lohne |
| Dr. med. Ilka Simon-Wagner | Praxis Dr. med. Ilka Simon-Wagner |
| Dr. med. Heiner Methfessel | Praxis Dr. Methfessel |
| Dr. med. Andree Allers | Kardiologie an der Paulikirche |
| Dr. med. Hermann Braun | Diabetespraxis Dr. Braun |
| Dipl.-med. Toralf Schwarz | Praxis T. Schwarz |
| Dr. med. Heidrun Täschner | Gemeinschaftspraxis Dr. Täschner / Dr. Bonigut |
| Dr. med. Sandra Schulz | Kardiologie/Internistenteam Kamen |
| Dr. med. Bernadett Brado | Praxis für Angiologie und Hämatologie |
| Dr. med. Daniel Zandt | Kardiologische Fachpraxis |
| Dr. med. Annette Birkenhagen | Praxis Dr. Birkenhagen |
| Dr. med. Cornelia Woitek | Praxis Dr. Woitek |
| Dr. med. Gabor Varnai | Praxis Dres. Jänsch / Varnai |
| Dr. Wolfgang Hartung | Kardiologische Praxis |
| Dr. Kurt Schwabe | Praxis am Landratspark |
| Dr. med. Andreas Schreckenberg | Dr. Schreckenberg |
| Dr. med Frank Warzok | Kardiologische Praxis |
| Dr. Nalan Coban | Kardiologie Potsdam |
| Dr. Martin Prohaska | Gemeinschaftspraxis Dr. med. Martin Prohaska und Dr. med. Felix Schulte |
| Mustafa Durak | Kardiologische Schwerpunktpraxis |
| Dr. Detlef Gysan | MVZ Gesundes Herz |
| Prof. Dr. med. Markus Zarse | Klinikum Lüdenscheid |
| Priv. Doz. Dr. med. Harilaos Bogossian | Evangelisches Krankenhaus Hagen-Haspe |
| Dr. Roy Ben-Chur | Praxis Dr. Ben-Chur |
| Dr. med. Thomas Walter | Praxis für Herzkreislauferkrankungen Greiz |
| Dr. Sabine Hansen | Praxis Dr. Sabine Hansen |
| Dr. med. Hans-Jürgen Ohler | Vivantes Klinikum Spandau |
| Dr. med. Alexander Krapivsky | Herzquartier Mühlheim, Kardiologische Gemeinschaftspraxis Dres. med. A. Krapivsky & P. Kekes |
| **Italy** |  |
| Prof. Marcello Arca | Policlinico Umberto I |
| Dr Matteo Pirro | A.O. Santa Maria della Misericordia |
| Prof. Francesco Giorgino | A.O.U. Consorziale - Policlinico Bari |
| Dott.ssa Patrizia Suppressa | A.O.U. Consorziale - Policlinico Bari |
| Prof. Francesco Cipollone | Ospedale SS. Annunziata |
| Prof. Claudio Ferri | Università dell'Aquila |
| Dr. Giovambattista Desideri | Presidio Ospedaliero San Filippo e Nicola |
| Dott.ssa Elena Alberghini | Ospedale Edoardo Bassini |
| Dr. Matteo Di Minno | A.O.U. Federico II |
| Prof. Ciro Mauro | A.O.R.N. Cardarelli |
| Dr Roberta Lupoli | A.O.U. Federico II |
| Dr. Antonio Pipolo | A.O.U. "San Giovanni di Dio e Ruggi d'Aragona" |
| Dr. Marco Lococo | Ospedale degli Infermi di Rivoli |
| Dr. Mauro Feola | Ospedale Regina Montis Regalis |
| Prof. Maurizio Averna | A.O.U. Policlinico "P. Giaccone" |
| Dr. Giovanni Licciardello | Ospedale E. Muscatello |
| Prof. Rossella Marcucci | A.O.U. Careggi |
| Prof. Claudio Borghi | A.O.U di Bologna-Policlinico S.Orsola-Malpighi |
| Dr. Roberto Catalini | Ospedale Macerata |
| Prof. Federico Guerra | Ospedali Riuniti "Umberto I - Lancisi - Salesi |
| Prof. Riccardo Sarzani | INRCA -Ospedale U. Sestilli di Ancona |
| Dr. Daniele Nassiacos | Presidio Ospedaliero di Saronno |
| Prof. Gianluca Calogero Campo | Arcispedale Sant’Anna Ferrara |
| Dr. Egidio Imbalzano | A.O.U. Policlinico "G. Martino" |
| Dr. Tiziano Angelo Lucchi | Fondazione IRCSS Ca' Granda Ospedale Maggiore Policlinico |
| Dr. Mariano Pellicano | Istituto Clinico Sant'Ambrogio |
| Dr. Gianni Casella | Ospedale Maggiore di Bologna |
| Dott.ssa Elena Repetti | Presidio ospedaliero Cardinal Massaia - A.S.L. AT |
| Dr. Michele Antonio Clemente | Presidio Ospedaliero Madonna Delle Grazie – A.S.M. Matera |
| Dr. Massimo Di Natale | Ospedale Santo Stefano di Prato - Azienda Usl Toscana centro |
| Dr. Gaetano De Ferrari | A.O.U. Città della Salute e della Scienza di Torino |
| Dr. Natale Daniele Brunetti | A.O.U. Ospedali Riuniti |
| Prof. Giovanni Esposito | A.O.U. Federico II |
| Dr. Giovanni Fazio | Casa di Cure Triolo Zancla |
| Dr. Paolo Calabrò | Azienda Ospedaliera di Rilievo Nazionale(A.O.R.N.) “Sant’Anna e San Sebastiano” di Caserta |
| Prof. Luca Dalle Carbonare | Ospedale Policlinico Rossi |
| Dott.ssa Antonia Alberti | Grande Ospedale Metropolitano di Niguarda |
| Prof. Edoardo Mannucci | A.O.U. Careggi |
| Prof. Agostino Gnasso | Azienda Ospedaliera Universitaria Mater Domini |
| Dr. Giuseppe Andò | A.O.U. Policlinico "G. Martino" |
| Dr. Francesco Amico | Ospedale Cannizzaro |
| Prof.ssa Giuseppina Novo | A.O.U. Policlinico "P. Giaccone" |
| Dr. Massimo Alessandri | Ospedale S. Andrea - Usl Toscana Sud Est |
| Dr. Andrea Di Lenarda | Azienda Sanitaria Universitaria Integrata di Trieste |
| Dott.ssa Tiziana Sampietro | Fondazione Toscana Gabriele Monasterio |
| Prof. Stefano Gonnelli | Azienda Ospedaliero-Universitaria Senese |
| Dr. Domenico D'Amario | Fondazione Policlinico Universitario A. Gemelli IRCCS |
| Dr. Claudio Bilato | Ospedale di Arzignano |
| Prof.ssa Livia Pisciotta | IRCCS Ospedale Policlinico San Martino |
| Prof.ssa Tiziana Montalcini | Azienda Ospedaliera Universitaria Mater Domini |
| Prof. Paolo Golino | A.O.R.N. Ospedale dei Colli |
| Dr. Marino Scherillo | A.O.R.N. San Pio - P.O. RUMMO |
| Prof. Giuseppe Boriani | A.O.U. Policlinico di Modena |
| Dr. Giovanni Luzzi | Presidio Ospedaliero Occidentale - Castellaneta |
| Prof. Ciro Indolfi | Azienda Ospedaliera Universitaria Mater Domini |
| Dr. Stefano Mazzarino | Ospedale Santo Spirito |
| Dr. Antonio Mugnolo | Ospedale Mater Salutis |
| Dr Elio Gorga | ASST Spedali Civili P.O di Brescia |
| Dott.ssa Alessandra Fiorentini | Ospedale Belcolle - ASL Viterbo |
| Prof. Guido Parodi | Ospedale Civile SS Annunziata |
| Prof. Raffaele De Caterina | Ospedale Cisanello - A.U.O.P. Azienda Ospedaliera Universitaria Pisana |
| Prof.ssa Maria Lorenza Muiesan | Azienda Socio Sanitaria Territoriale degli Spedali Civili di Brescia |
| Dr. Daniele Andreini | Centro Cardiologico Monzino - IRCCS |
| Dr. Alessandro Navazio | Arcispedale Santa Maria Nuova - Azienda USL/IRCCS Reggio Emilia |
| Dott.ssa Mila Straniti | Ospedale SS Cosma e Damiano di Pescia - Azienda Usl Toscana centro |
| Prof.ssa Maria Del Ben | Azienda Ospedaliero Universitaria - Policlinico Umberto I |
| Prof.ssa Francesca Carubbi | Ospedale Civile di Baggiovara- AOU di Modena |
| Dr. Daniela Aschieri | Ospedale Unico della Valtidone |
| Dr. Claudio Fresco | Presidio Ospedaliero "Santa Maria della Misericordia" di Udine |
| Dr. Massimo Grimaldi | Ente Ecclesiastico Ospedale Generale Regionale “F.Miulli” |
| Dr. Josè Pablo Werba | Centro Cardiologico Monzino - IRCCS |
| Prof. Roberto Trevisan | Azienda Ospedaliera Papa Giovanni XXIII |
| Dr. Antonino Nicosia | Ospedale Giovanni Paolo II |
| Dott.ssa Donata Angela Mor | Fondazione Poliambulanza Istituto Ospedaliero - Brescia |
| Dr. Emilio Di Lorenzo | A.O. San Giuseppe Moscati |
| Dr. Natale Di Belardino | ASL Roma 6 - Ospedali Riuniti Anzio – Nettuno |
| Prof.ssa Angelina Passaro | Azienda Ospedaliera-Universitaria S. Anna di Ferrara |
| Prof.ssa Anna Solini | Università di Pisa |
| Dr. Antonio Lanzilli | A.O. San Giuseppe Moscati |
| Dr. Vittorio Salvatore | Ospedale Civile Santa Maria Incoronata dell'Olmo |
| Dr. Giuseppe Colonna | Presidio Ospedaliero Vito Fazzi |
| Dr. Alessandro Lupi | Ospedale dei Castelli |
| Prof. Carlo Di Mario | A.O.U. Careggi |
| Dr. Luigi Di Lorenzo | Ospedale Civile San Rocco |
| Dott.ssa Ilaria Jacomelli | Ospedale Policlinico Casilino |
| Dr. Giovanni Paternò | Ospedale San Carlo |
| Dr. Giuseppe Mandraffino | Azienda Ospedaliero Universitaria Policlinico "G. Martino" |
| Dr. Giovanni Tortorella | Ospedale Vaio Fidenza |
| Dott.ssa Marta Focardi | Ospedale Le Scotte |
| Dr. Giuseppe De Blasio | IRCCS Istituto Ortopedico Galeazzi |
| Dr. Cinzia Vespucci | Ospedale Abbadia San Salvatore |
| Dr. Roberto Cemin | Ospedale di Bolzano |
| Dott.ssa Elena Ferdenzi | Ospedale Guglielomo da Saliceto |
| Dott.ssa Lucia Filippucci | USL Umbria I |
| Dott.ssa Liliana Grigore | IRCCS Multimedica |
| Dr. Lorenzo Maroni | Ospedale di Gallarate - ASST Valle Olona |
| Dr. Paolo Midi | Ospedale dei Castelli - ASL Roma 6 |
| Prof. Giorgio Sesti | Azienda Ospedaliero - Universitaria Sant'Andrea |
| Dr. Emilio Di Vincenzo | Ospedale di Prato |
| Prof. Francesco Prati | Azienda Ospedaliera San Giovanni Addolorata |
| Dr. Michele Comito | Ospedale G. Jazzolino |
| Dr. Alessandro Sciahbasi | Ospedale Sandro Pertini - ASL Roma 2 |
| Dr. Franco Bui | Ospedale di Nottola- Azienda Usl Toscana Sud Est |
| Prof. Francesco Grigioni | Policlinico Universitario Campus Bio-Medico |
| Dr. Gianfranco Tortorici | Ospedale di Bentivoglio |
| Dr. Vito Sollazzo | Presidio Ospedaliero Giuseppe Tatarella |
| Dr. Mirza Becirovic | Ospedale di Carpi |
| Dr. Giuseppe Caramanno | Ospedale San Giovanni di Dio |
| Prof.ssa Adriana Branchi | Fondazione IRCSS Ca' Granda Ospedale Maggiore Policlinico |
| Prof. Dario Manfellotto | Ospedale San Giovanni Calibita FateBeneFratelli - Isola Tiberina |
| Dott. Fabio Menghini | Ospedale Sant'Eugenio - ASL Roma 2 |
| Dott. Nazzareno Galiè | Policlinico Sant'Orsola-Malpighi |
| Dott. Massimo Puato | Presidio Ospedaliero di Mirano - ULSS 3 Serenissima |
| Prof.ssa Adriana Visonà | Ospedale di Castelfranco |
| Dr. Filippo Sarullo | Ospedale Buccheri La Ferla |
| Prof. Carmine Vecchione | A.O.U. San Giovanni di Dio e Ruggi d’Aragona |
| Prof. Raffaele Marfella | Primo Policlinico di Napoli - Università degli Studi della Campania Luigi Vanvitelli |
| Dott.ssa Anna Dell'Elce | Ospedale San Giuseppe |
| Dott.ssa Annamaria Nicolino | Ospedale Santa Corona |
| **Netherlands** |  |
| Dr Martijn van Eck | Jeroen Bosch Ziekenhuis |
| Dr. Gerard Linssen | Ziekenhuis Groep Twente (ZGT) |
| Dr Bas Hamer | Meander Medical Center |
| Dr. Bjorn Groenemeijer | Gelre Ziekenhuis |
| Dr Driek Beelen | IJsselland Ziekenhuis |
| Dr. Maarten Asselman | Ziekenhuis Rivierenland (ZRT) |
| Dr Karin Arkenbout | Tergooi |
| Dr Johanna Drost | Saxenburgh Medisch Centrum |
| Dr Fabrice Martens | Deventer Ziekenhuis |
| Dr Cornelis de Nooijer | Maxima Medical Centre (MMC) |
| Dr. Dirk Schellings | Slingeland Ziekenhuis |
| Dr. Stijntje Zoet-Nugteren | Ikazia Ziekenhuis |
| Prof Dr Frank Visseren | University Medical Center Utrecht (UMCU) |
| Dr Ismail Aksoy | Admiraal de Ruyter Ziekenhuis (ADRZ) |
| Prof Dr Wouter Jukema | Leids Universitair Medisch Centrum (LUMC) |
| Dr Clara van Ofwegen-Hanekamp | Diakonessenhuis Utrecht |
| Dr Jan Constandse | Reinier de Graaf Gasthuis (RDGG) |
| Dr. Thomas Oosterhof | Ziekenhuis Gelderse Vallei |
| Dr Pieter Dijkmans | Zaans Medisch Centrum |
| Dr Robert van Es | Treant Zorggroep |
| Dr Aaf Kuijper | Spaarne Gasthuis |
| Dr Arnout Haasdijk | Haga Ziekenhuis |
| Dr Eric Viergever | Groene Hart Ziekenhuis |
| Dr Jeroen van Etten | BovenIJ ziekenhuis |
| Dr Marc van der Zee | St Jansdal |
| Dr. Walter Hermans | ETZ |
| Dr Matthijs Meijs | Medisch Spectrum Twente |
| Dr Ka Wai Wu | Het van Weel - Bethesda |
| Dr Ruud van de Wal | Bernhoven |
| **Portugal** |  |
| Dr. Carlos Aguiar | Instituto do Coração, ICSM, S.A |
| Prof. Dr. Pedro Monteiro | Clínica Cuida Mais |
| Dr. Jorge Mimoso | Centro Hospitalar Univesitario do Algarve |
| Dr. João Sequeira Duarte | Hospital Egas Moniz (CHLO) |
| Prof. Dr. Fausto Pinto | Centro Hospitalar Universitário Lisboa Norte - Hospital Santa Maria |
| Prof. Dr. Patrício Aguiar | Centro Hospitalar Universitário Lisboa Norte - Hospital Santa Maria |
| Prof. Dr. João Raposo | Associação Protectora dos Diabéticos de Portugal |
| Dr. Fernando Pinto | Centro Hospitalar de Entre Douro e Vouga |
| Prof. Dr. Davide Carvalho | Centro Hospitalar Universitário São João (CHUSJ) |
| Prof. Dr. Pedro von Hafe | Instituto CUF Porto |
| Dr. Paulo Alves | H. Lusíadas de Lisboa |
| **Republic of Ireland** |  |
| Professor Richard Sheahan | Beaumont Hospital |
| Dr Ross Murphy | St. James' Hospital |
| Dr David Burke | Beacon Hospital |
| Dr Eugene McFadden | Cork University Hospital |
| Professor Robert Byrne | Mater Private Hospital |
| Dr Dermot Nolan | Turloughmore health Centre |
| **Spain** |  |
| Dr. José María Mostaza | Hospital Universitario La Paz - Carlos III |
| Dr. Carlos Lahoz | Hospital Universitario La Paz - Carlos III |
| Dr. Pedro Chinchurreta | Hospital Costa del Sol |
| Dra. Iria Pinal | Complejo Hospitalario Universitario de Ourense (CHOU) |
| Dr. Manuel Martínez-Sellés | Hospital Universitario Gregorio Marañon |
| Dra. Beatriz Pérez Villardón | Hospital Regional Universitario de Málaga |
| Dr. Luis Garcia Ortiz | Centro de Salud de la Alamedilla |
| Dr. Santiago Camacho Freire | Hospital de San Agustín |
| Dr. Javier Chimeno García | Complejo Asistencial de Zamora |
| Dr. Abel Garcia del Egido | Hospital Universitario de León |
| Dr. Antonio Miguel Barragán Acea | Clinica Privada Orotava |
| Dra. Miriam García Bermudez | Hospital de Sant Joan Despí Moisès Broggi |
| Dr. José Luis Moriñigo Muñóz | Hospital Clínico de Salamanca |
| Dr. Pedro Valdivieso | Hospital Virgen de la Victoria |
| Dr. Carlos Pérez Muñoz | Consulta Particular de Cardiología |
| Dr. Javier León | Hospital Universitario de Jérez de la Frontera |
| Dr. Jordi Puntí Sala | Hospital Parc Tauli |
| Dr. Manuel Jesús Romero Jimenez | Hospital Infanta Elena |
| Dr. Jose Manuel Rubio Campal | Fundación Jiménez Díaz |
| Dr. Eddie Velásques | Hospital HM de Torrelodones |
| Dr. Antonio José Fernández Romero | Hospital de Alta Resolución de Utrera |
| Dr. Saul Suarez | Centro de Salud Colloto |
| Dr. Fernando Gómez Peralta | Hospital General de Segovia |
| Dr. Xavier Pinto Sala | Hospital Universitari de Bellvitge |
| Dra. Vanessa Escolar Pérez | Hospital Universitario de Basurto |
| Dra. Amaia Ibarra Gutierrez | Hospital San Eloy |
| Dra. Olga Quiñones | Centro de Salud el Cristo |
| Dr. Carlos Arias Miranda | Hospital de Alta Resolución de Lebrija |
| Dr. Joaquin Aracil | Centro de Salud el Cristo |
| Dr. Consuelo Olivo | Hospital de Alta Resolución de La Janda |
| Dra. Isabel Belloso | Centro de Salud Reboleria |
| Dr. Francisco Gómez Delgado | Hospital Universitario Reina Sofia |
| Dr. Victor León Arguero | Hospital Universitario Central de Asturias |
| Dr. Juan Pedro Botet Montoya | Hospital del Mar |
| Dr. Domingo Pascual Figal | Hospital Clínico Virgen de la Arrixaca |
| Dr. Antonio Gómez Menchero | Hospital Juan Ramón Jiménez |
| Dra. Mónica Domenech | Hospital Clínic i Provincial de Barcelona |
| Dra. Raquel Solanilla Rodríguez | Hospital de Alta Resolución de Sierra Norte |
| Dra. Iris Maria Esteve Ruiz | Hospital de Alta Resolución de Écija |
| Dr. Antonio Pérez | Hospital de la Santa Creu i Sant Pau |
| Dr. Reyes Oliva Encabo | Hospital Universitario de Móstoles |
| Dr. Pedro Talavera | Hospital Universitario de Fuenlabrada |
| Dra. Elvira Blanco | Complejo Hospitalario de Ourense |
| Dr. Fernando Alfonso Manterola | Hospital Universitario de la Princesa |
| Dr. Jacinto Fernández | Hospital General Reina Sofia |
| Dra. Teresa Arrobas | Hospital Universitario Virgen de la Macarena |
| Dr. Carlos Escobar | Hospital Universitario La Paz - Carlos III |
| Dr. Nuria Plana | Hospital Sant Joan de Reus |
| Dr. Luis Manzano | Hospital Universitario Ramón y Cajal |
| Dra. Iluminada García Polo | Hospital Universitario la Princesa |
| Dr. José Luis Diaz Diaz | Complejo Hospitalario Universitario de A Coruña (Hospital Abente y Lago) |
| Dr. Moises Rodriguez Mañero | Hospital Clínico Universitario de Santiago de Compostela |
| Dr. Sonia Ruiz Bustillo | Hospital del Mar |
| Dr. Juan de Dios García Diaz | Hospital Universitario Príncipe de Asturias |
| Dr. Carlos Guijarro Herraiz | Hospital Universitario Fundación Alcorcón |
| Dr. Fernando Civeira Murillo | Hospital Universitario Miguel Servet |
| Dr. Francisco Martínez Debén | Hospital Naval de Ferrol |
| Dr. Leopoldo Pérez de Isla | Hospital Clínico Universitario San Carlos |
| Dr. Juan Diego Mediavilla García | Hospital Universitario Virgen de las Nieves |
| Dr. Juan Carlos Pérez Marin | Complejo Hospitalario Universitario Insular |
| Dr. Manuel García de Yébenes Castro | Clinica Universitaria de Navarra |
| Dra. Mónica Marazuela | Hospital Universitario la Princesa |
| Dr. Rafael Simó | Hospital Universitario Vall d'Hebron |
| Dr. Rafael Ramírez Montesinos | Hospital Sant Pau i Santa Tecla |
| Dra. Isabel Pavón de Paz | Hospital Universitario de Getafe |
| Dr. Cristina García Abreu | Hospital Royo Villanova |
| Dra. África García Roy | Centro de Salud Fuentes Norte |
| Dr. Raimundo Andres Cañas | Fundación Jiménez Díaz |
| Dr. Carles Jericó | Hospital Sant Joan Despí-Moisés Broggi |
| Dr. José Luis Zamorano | Hospital Universitario Ramón y Cajal |
| Dra. Antonia Espinosa Collado | Centro de Salud Villa de Otura |
| Dr. Javier Espiga Alzola | Hospital de Basurto |
| Dr. José Luis Hernandez Hernandez | Hospital Universitario Marqués de Valdecilla |
| Dr. Jorge Francisco Gómez Cerezo | Hospital Universitario Infanta Sofía |
| Dr. Agustín Blanco Echevarría | H. Universitario 12 de octubre |
| Dr. Antonio Garcia Quintana | H. Universitario Gran Canaria Dr. Negrín |
| Dr. Alberto Esteban | Hospital Universitario Severo Ochoa |
| Dr. Manuel Suárez Tembra | Hospital San Rafael |
| Dr. Santiago Freire | Complejo Hospitalario Universitario de A Coruña |
| **Sweden** |  |
| Professor Mats Eriksson | Karolinska University Hospital, Huddinge |
| MD Carl-Johan Lindholm | Clemenstorgets Hjärtmottagning |
| GP Andrzej Sloma | Värmdö Vårdcentral, |
| Professor, MD Anders Gottsäter | Skåne University Hospital |
| Professor Stefano Romeo | Sahlgrenska University Hospital |
| Professor Mikael Dellborg | Östra Sahlgrenska Universitetssjukhuset – Ostra Sjukhuset |
| MD Nils Witt | Södersjukhuset Stockholm |
| Dr. Neshro Barmano | Länssjukhuset Ryhov |
| Dr. Peter Hallgren | Falu Lasarett, Diab/Endo-mottagning |
| Dr. Staffan Salmonsson | Öbackakliniken |
| Dr. Christos Milonas | Cityheart |
| **Switzerland** |  |
| Dr. David Nanchen | University of Lausanne Unisanté |
| Dr. Konstantinos Koskinas | Inselspital Universitätsspital Bern |
| Prof. Georg Ehret | Hôpitaux Universitaires de Genève |
| Prof. Dr. med. Isabella Sudano | Universitätsspital Zürich |
| Prof. Christian Müller | Universitätsspital Basel |
| Dr. Michael Egloff | Kantonsspital Baden AG |
| PD Dr. Stefan Bilz | Kantonsspital St. Gallen |
| Prof. Dr. med. Christophe Wyss | Klinik Hirslanden |
| Prof. Dr. André Roger Miserez | Diagene Research Institute |
| PD Dr. med. Florim Cuculi | Cardio Center Luzern |
| Dr. méd. Stéphane Bosquet | Cardiologue FMH |
| Prof. Dr. med. Gottfried Rudofsky | Solothurner Spitäler AG |
| Dr. med. Michel Romanens | Kardiolab |
| **United Kingdom** |  |
| Dr Samir Purnell Mullick | The Atherstone Surgery |
| Dr Damien McNally | Ormeau Clinical Trials Ltd |
| Dr Anthony Gunstone | Staploe Medical Centre |
| Dr John Ryan | The Alverton Practice |
| Dr Jonathan Garstang | Knowle House Surgery |
| Dr Nick Jacobsen | Newquay Health Centre |
| Dr Andrew Moriarty | Craigavon Area Hospital |
| Dr Wayne Turner | The Burbage Surgery |
| Prof Terry McCormack | Whitby Group Practice |
| Dr Tim Johnson | Heart of Bath Medical Partnership |
| Dr Matthew Capehorn | Clifton Medical Centre |
| Dr Chris Schramm | Wansford and Kings Cliffe Practice |
| Dr Paul Guyler | Southend University Hospital |
| Dr Deepa Narayanan | Hull Royal Infirmary |
| Dr Graham Toms | Oak Tree Surgery |
| Dr Paul Ainsworth | Sherbourne Medical Centre |
| Dr Margaret Ikpoh | Holderness Health Surgery |
| Dr Ahmet Fuat | Carmel Medical Practice |
| Dr Jon Dickson | Ecclesfield Group Practice |
| Dr Nigel Capps | Princess Royal Hospital |
| Dr Karthik Viswanathan | Huddersfield Hospital |
| Dr Stewart Pattman | North Tyneside General Hospital |
| Dr Satheesh Balakrishnan-Nair | Glan Clwyd Hospital |
| Dr Angela Gbegbaje | Royal Albert Edward Infirmary |
| Dr Manish Saxena | Barts Health NHS Trust |
| Dr Christopher Keast | The Boathouse Surgery |
| Dr Duncan Browne | Royal Cornwall Hospital |
| Dr Azhar Zafar | Danes Camp Medical Centre |
| Prof Gregory Lip | Liverpool Heart & Chest Hospital NHS Trust |
| Dr Patrick Moore | The Adam Practice |
| Dr Stacey Fisher | Carlisle Healthcare |
| Dr Stacey Fisher | Fellview Healthcare |
| Dr Stacey Fisher | Temple Sowerby Medical Practice |
| Dr Gareth Powell | Llanedeyrn Health Centre |
| Dr Tamsin Sevenoaks | Brockwood Medical Practice |
| Dr Tom Hyde | Great Western Hospital Swindon |
| Dr Peter Carey | Sunderland Royal Hospital |
| Dr Mike Butler | Waterloo Medical Care |
| Dr Cyril Evbuomwan | Church End Medical |
| Dr S Thiagu | Pickering Medical Practice |
| Dr Ian Schofield | Panthera Preston |
| Dr Gordon Irvine | Breckland alliance |
| Dr Farzan Kamali | Branch End Surgery |
| Dr Stephanie Hughes | The Friarsgate Practice |
| Dr John Wakeling | Ely Bridge Surgery |
| Dr Samir Purnell Mullick | The Atherstone Surgery |
